# Southern Right Whale (*Eubalaena australis*) Reproductive Success is Influenced by Krill (*Euphausia superba*) Density and Climate

**DOI:** 10.1038/srep28205

**Published:** 2016-06-16

**Authors:** Elisa Seyboth, Karina R. Groch, Luciano Dalla Rosa, Keith Reid, Paulo A. C. Flores, Eduardo R. Secchi

**Affiliations:** 1 Universidade Federal do Rio Grande, Instituto de Oceanografia, Laboratório de Ecologia e Conservação da Megafauna Marinha, Rio Grande, CEP 96201-900, Brazil; 2 Projeto Baleia Franca, Centro Nacional de Conservação da Baleia Franca, Imbituba, CEP 88780-000, Brazil; 3 Commission for the Conservation of Antarctic Marine Living Resources, Hobart, 7000, Australia; 4 Centro Mamíferos Aquáticos, Instituto Chico Mendes para Conservação da Biodiversidade, Ministério do Meio Ambiente, Florianópolis, CEP 88053-700, Brazil

## Abstract

The reproductive success of southern right whale (*Eubalaena australis*) depends on body condition and, therefore, on foraging success. This, in turn, might be affected by climatically driven change in the abundance of the species main prey, krill (*Euphausia superba*), on the feeding grounds. Annual data on southern right whale number of calves were obtained from aerial surveys carried out between 1997 and 2013 in southern Brazil, where the species concentrate during their breeding season. The number of calves recorded each year varied from 7 to 43 (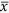 = 21.11 ± 11.88). Using cross-correlation analysis we examined the response of the species to climate anomalies and krill densities. Significant correlations were found with krill densities (r = 0.69, p = 0.002, lag 0 years), Oceanic Niño Index (r = −0.65, p = 0.03, lag 6 years), Antarctic Oscillation (r = 0.76, p = 0.01, lag 7 years) and Antarctic sea ice area (r = −0.68, p = 0.002, lag 0 years). Our results suggest that global climate indices influence southern right whale breeding success in southern Brazil by determining variation in food (krill) availability for the species. Therefore, increased frequency of years with reduced krill abundance, due to global warming, is likely to reduce the current rate of recovery of southern right whales from historical overexploitation.

Climate variability has strong effects on marine ecosystems, from scales that impact individuals to those that impact the entire food web^1^,^2^. For individuals and populations, climate effects may be direct, through physiology, involving metabolic and reproductive processes, or indirect, through the ecosystem. The latter includes the interactions between prey, predators and competitors through the impact on food availability and species distribution^3^.

The biological effects of El Niño have been extensively studied in marine ecosystems^4^,^5^,^6^. El Niño is defined by the appearance and persistence, for more than 5 months, of anomalously warm water in the coastal and equatorial ocean off western South America^7^. This phenomenon has been shown to have a great potential to alter marine ecosystems through marked increase in ocean temperature^3^.

Observations of anomalies that occurred during El Niño events have demonstrated the global connection of weather system as well as showing how anomalies can be transmitted from the Pacific to other ocean basins through ocean-atmosphere interactions. Such teleconnections were found between Pacific El Niño regions and South Georgia, in the Southern Ocean, in relation to sea surface temperature (SST). The strongest correlation observed was between the signal in the west Pacific (El Niño 4 region) and the SST around South Georgia with a delay of approximately three years^8^. The manifestation of these physical effects can subsequently be seen in biological effects, often illustrated by the response of upper-trophic level predators^9^,^10^.

The Antarctic krill (*Euphausia superba*) is a key component of the food web in the Southern Ocean given its large biomass^11^, which has strong association with physical processes including the extent and duration of sea-ice^12^. In waters around South Georgia, krill abundance declined when SST was higher than normal, in association with El Niño events^13^. This decline was attributed to the negative impact of warmer water on the recruitment of krill^14^. As a consequence of the reduction in krill abundance the reproductive performance of many krill-dependent marine mammals and seabirds was dramatically reduced^9^,^10^,^15^,^16^,^17^,^18^.

The effects of reduced food availability in marine systems are typically documented with respect to changes in the performance of land-based marine predators. This reflects the relative ease of monitoring of land-based species during periods when they are constrained to return to a central location to provision offspring. For other marine species that are not central-place foragers, such as cetaceans, there is evidence that reproduction can also be suppressed during periods of nutritional stress^19^,^20^,^21^,^22^,^23^. For females, nutrition is fundamental in determining the age of sexual maturity, ovulation, fertility, quality and quantity of milk production, and birth interval. Body condition might affect ovulation and is crucial mainly in the period prior to pregnancy of both southern (*Eubalaena australis*) and northern (*Eubalaena glacialis*) right whales^24^. This relationship seems to be regulated by the hormone leptin, produced by adipose tissue that acts in hypothalamus and pituitary to stimulate the secretion of gonadotropin-release hormone and luteinizing hormone, respectively^25^. Furthermore, nutrition during gestation is fundamental to fetal development as well as offspring survival and growth^20^.

Although few studies have examined southern right whale diet, krill seems to be an important item for the species^26^,^27^,^28^. Within the Atlantic Ocean, the southern right whale breeding population of Península Valdés, Argentina, produces fewer calves than expected following years of higher SST anomalies around South Georgia (its presumed feeding ground) and El Niño 4 region^29^. Given the connections between the population of southern right whale that reproduces on the Brazilian coastal waters to that in Argentina^30^ and evidence of culturally inherited site fidelity to feeding grounds of this species^28^, it is reasonable to assume that the whales from the two populations feed in same area of the Southern Ocean during summer. If this assumption holds, it can be expected that changes in krill abundance near South Georgia would cause variation on the birth rates of southern right whales breeding off Brazil.

Therefore, the objective of this study was to investigate the influence of krill abundance, as well as climate and oceanographic factors that influence krill abundance, on the reproductive success of southern right whale population breeding off southern Brazil. The hypothesis to be tested is that krill abundance in the region near to South Georgia is correlated with observed southern right whale calf production in its main breeding ground off Brazil.

## Methods

**Figure 1:**
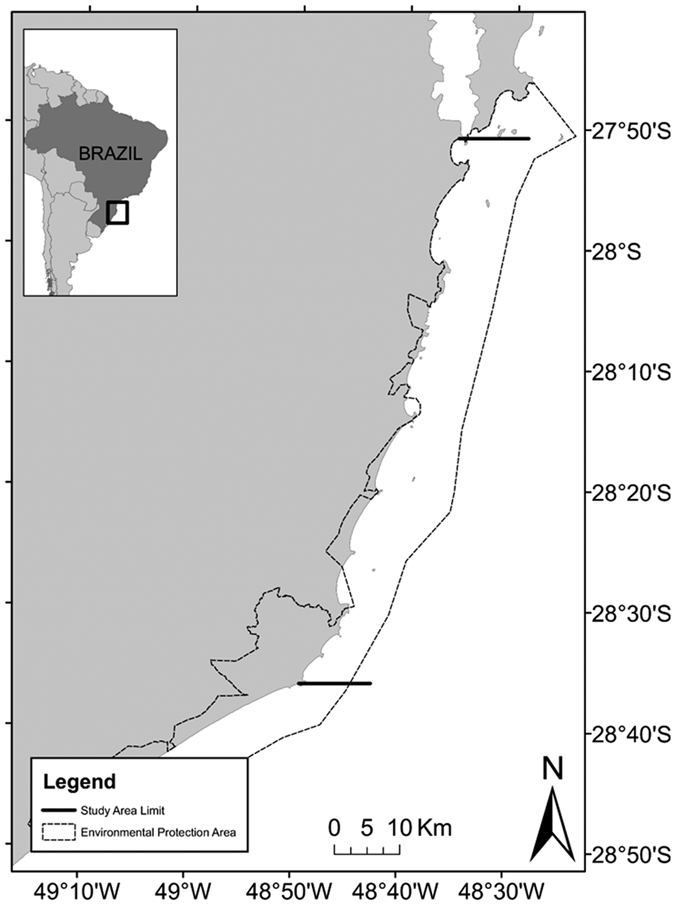
Map of the studied area in Santa Catarina, Brazil. The monitored area (limited by the black lines) is included in the Environmental Protection Area (APA da Baleia Franca) created specifically to protect the southern right whale, *Eubalaena australis*. Image created using QGIS software version 2.8.2 (www.qgis.org).

Data on the number of southern right whale calves has been obtained through annual aerial surveys carried since 1987. The total area monitored varied between years, but an area between Cabo de Santa Marta (28°36′35″S) and Pântano do Sul beach (27°53′00″S) was regularly monitored from 1997–2013 and is considered the study area (Fig. 1). This area holds the highest breeding concentration of southern right whale in Brazil and the second largest in the western side of the Southern Atlantic. It is located within an environmental protection area (Brazilian Government Federal Decree of 14 September 2000) that was created to manage human activities in a region that is crucial to the life cycle of southern right whales. In many years, more than one flight was conducted during the breeding season of southern right whales, but only data from the flight closest to the peak of sightings during this period (September) were considered.

Flights were carried out with the purposes of estimating the number of southern right whales present in the region during their reproductive season and photo-identifying individuals. In the present study, photo-identification data were only used to minimize chances of double countings. A single-engine aircraft was used in 1997 and a helicopter in the following years. All flights followed a parallel trajectory at a distance of approximately 500 m from shore and at a tentative altitude of 300 m. The search for individuals was restricted to a stripe of up to 1500 m from shore, where mother-calf pairs concentrate^31^.

Two to three observers were involved in the search duty and data recording. The main observer sat next to the pilot and searched for whales ahead of the aircraft. The second observer sat behind the pilot and scanned to the right of the aircraft and was responsible for taking photographs from the sighted animals. The third observer sat next to the photographer and monitored the area to the left of the aircraft. This side was also observed by the main observer when only two observers were onboard. It was assumed that this variation in the number of observers would not affect the estimates of calf number. Surveys were carried out during favorable sighting conditions with good visibility and sea state below 3 in Beaufort scale. When a sighting occurred, the aircraft approached the whale(s) to a least distance of 100 m for counting and photo-identifying the individuals.

The aircraft circled the whale groups sighted and the helicopter hovered over them at a minimum height of 100 m, in accordance with approved regulations (Federal Law 7.643/1987). Approaches were halted if it appeared to change the whale’s behavior.

Number of individuals, group composition and their location (determined using a Global Positioning System – GPS) were recorded for each sighting. The individuals were classified as adults, juveniles or cow-calf pairs. For the purposes of this study, we considered juveniles as adults. We used the number of observed calves as a measure of the reproductive success. The population size of southern right whales is considered to be recovering following the cessation of commercial whaling (whaling ceased in 1973 in Brazil)^32^,^33^ and the count data on the number of calves was expected to show a positive trend over time. In order to account for the influence of such a trend on the correlation analyses data were detrended (Table 1) by subtracting a least-squares-fit straight line^34^ using R software version 3.1.1^35^.

Estimates of krill densities for South Georgia adjacencies (data extracted from previous study)^36^ were used to assess the effect of food availability on southern right whale reproductive success. As data on krill density were missing for 2008, four alternative values were used instead to assess their influence on the analysis output: i. the mean between 2007 and 2009 densities; ii–iv. the lower, the mean and the higher densities available in the dataset. As no difference was observed in the output, the first alternative value was used to replace the missing data.

The effects of climate anomalies were evaluated using data from climate indices (Oceanic Niño Index–ONI and Antarctic Oscillation- AAO), SST anomalies around South Georgia (SSTSG) and sea ice anomalies. Climate indices were obtained from the database available from NOAA Climate Predict Center (http://www.cpc.ncep.noaa.gov), while the South Georgia SST values were obtained from Kaplan database (http://wwwesrl.noaa.gov/). We used sea ice area anomalies for the Antarctic-wide area (http://nsidc.org/data/seaice_index/) and for Weddell Sea area (ftp://sidads.colorado.edu/pub/DATASETS/nsidc0192_seaice_trends_climo/total-ice-area-extent/nasateam/), referred as SIAA and SIAW, respectively. This last locality was included because it is considered the source of Antarctic krill for the South Georgia region^37^. As in previous studies dealing with teleconnections^29^,^38^, for each variable the value was averaged from June of the previous year to May of the year that is being considered. Variables such as Southern Oscillation Index (SOI) and SST anomalies from El Niño 4 region (both obtained from http://www.cpc.ncep.noaa.gov), presented significant collinearity with other variable and were, therefore, excluded from the analysis.

Relationships between the detrended number of calves in each year and the selected covariates were examined through cross-correlation analyses^39^ using the PAST software. The method of cross-correlation is used to identify the time lag that maximizes the correlation between the explanatory and target variables^39^. Separately, the potential correlation of each covariate and the detrended number of southern right whale calves was tested. Only datasets that satisfied the recommendation regarding cross-correlation analyses^39^, i.e. containing at least two complete cycles of each variable being tested, were included in the analysis. The correlation between variables and the lag between them was considered significant for the 95% confidence interval. Given that time series correlations are complex and easily subject to spurious correlation, essentially because in conducting multiple tests it changes the requirements for significance, we performed a simple correlation test between each of the two explanatory variables with zero time-lag (i.e. krill density and SIAA) and the detrended number of southern right whale calves.

## Results

The number of calves for each year were based on the 830 sighting occasions and varied from 7–43 (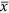
=21.11 ± 11.88) (Table 1).

**Figure 2:**
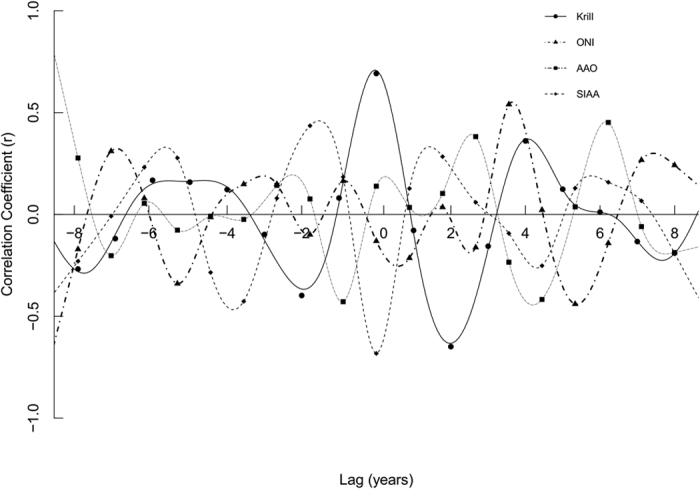
Correlation between detrended number of southern right whale calves and lagged variables indicated as significantly influencing on it. Krill = krill densities near South Georgia, ONI = Oceanic Niño Index, AAO = Antarctic Oscillation and SIAA = Antarctic sea ice area. Image created using R software version 3.1.1 (www.R-project.org).

**Figure 3:**
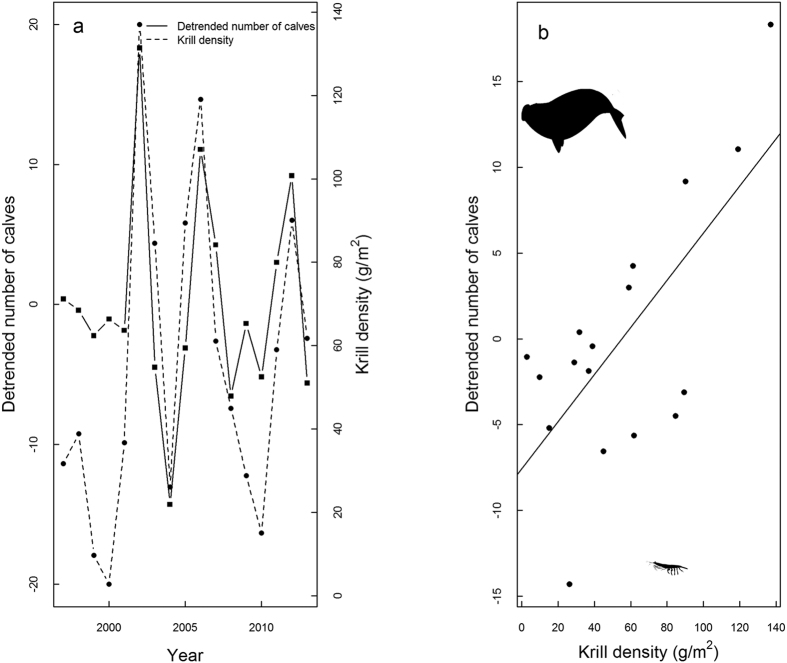
Detrended number of southern right whale calves and krill density (g/m^2^) around South Georgia variations during the study period, from 1997 and 2013 (**a**) and correlation between the detrended number of calves on the Brazilian breeding ground and krill density (**b**). Image created using R software version 3.1.1 (www.R-project.org).

Significant correlations were found between the detrended number of calves and krill densities, ONI, AAO and SIAA for different time lags (Table 2; Fig. 2). Simple correlation confirmed the strong relationship between the detrended number of calves and SIAA (r^2^ = −0.46) and krill densities (r^2^ = 0.48) (Fig. 3). Correlations with SIAW and SSTSG were non-significant.

## Discussion

The positive correlation between the number of southern right whale calves in southern Brazil and krill densities near South Georgia, considered an important feeding ground for this whale population, suggests that the reproductive success of southern right whale is directly influenced by food availability during the early months of gestation.

Although this is a 0-yr lag for the correlation of krill densities and number of calves, it is important to recognize that this actually reflects a lag of 9 months between the measurement of krill densities (typically in January/February) and the survey of the number of calves in September/October. A relationship at a similar time scale was found between sea ice data (used as a proxy of *E. superba* abundance) and body condition of humpback whales (*Megaptera novaeangliae*) caught in west Australian waters^40^. Variable time-lag correlation between feeding conditions and nutritional status have also been found in northern right whales (*Eubalaena glacialis*) where calving rates and the abundance of the copepod *Calanus finmarchicus* (the main food of those whales) were significantly correlated at a 0 yr and a 2 yr lag, depending on the time period analyzed^21^. For bowhead whales (*Balaena mysticetus*) in Alaskan and Canadian waters, body condition was highly correlated to environmental settings that influence food availability in the preceding summer as well as in three summers earlier^41^.

Thus it is apparent that nutrition can influence different phases of reproduction, such as conception, implantation, gestation and lactation, with consequences for calf survival^20^,^42^. Therefore it is possible that the required body condition for reproduction is reached through cumulative energy storage over successive years of abundant krill supply such that a single good year of food availability for the whales might not be sufficient to provide the necessary nutritional condition if the individuals had experienced one or more previous years of poor krill supply. The reproductive cycle of southern right whales lasts, on average, three years, one for gestation, one for lactation and the last one to recover and build fat reserves for the next pregnancy^43^,^44^,^45^. Thus it is clear that feeding conditions at all stages in the cycle may determine the likelihood of reproductive success for an individual animal. Given the prolonged nature of the reproductive cycle in baleen whales this has the potential to introduce variable lags between periods of nutritional stress and subsequent reproductive output^21^. However, the results of the present study strongly suggest that nutritional conditions during gestation have a significant impact on the subsequent calving rates of southern right whales. Such observation was made for southern right whale population observed in Península Valdés, Argentina, for which feeding conditions in the summer of pregnancy were associated to the duration of the reproductive cycle^46^.

The krill population at South Georgia is not self-sustaining, being dependent upon input from areas further south on the Scotia Arc and the Weddell Sea transported by the Antarctic Circumpolar Current. Therefore, the amount of krill reaching South Georgia reflects an interaction between changes in krill production in those source regions as well as changes in oceanic circulation that transport the krill to South Georgia^37^. In addition, the time it takes for SST anomalies to influence krill densities in the region may vary from a few months to two years, depending on their intensity^37^,^47^.

It is apparent that the relationship between climate indices originating in the Pacific, sea ice variability in the Antarctic, krill abundance at South Georgia and the breeding success of southern right whales in Brazil is inherently complex. However, the 6-year time-lag in the relationship between calf production and ENSO signals are broadly consistent with a series of physical and biological teleconnections linking two of the major oceans. The time-lag between elevated SST at west Pacific (El Nino 4 region) and at South Georgia was of the order of 3 years; these periods of elevated SST at South Georgia are associated with a reduction in krill recruitment in that year, which is manifested in a reduction in the biomass of krill in the following summer^48^,^49^ and those krill densities at South Georgia in January/February were highly correlated with the calf production in Brazil 9 months later.

Although there is evidence of a large-scale reduction in krill biomass in previous decades in the southwest Atlantic^50^, the time-series used in the present study^36^ does not suggest that such a decline has continued. However, authors in a previous study^14^ used regression models that related future climate change scenarios with krill density and postulated that in a scenario of an increase in 1 °C over the next 100 years there would be a 95% reduction in krill biomass within 50–60 years. If global climate conditions continue to change, following actual warming projections^51^,^52^, these stocks could be prevented from overcoming years of low recruitment, and then continue to decline, with stronger influence on southern right whale population dynamics. Given the strength of the relationship between the abundance of krill at South Georgia and southern right whale productivity in the southwest Atlantic Ocean, it would seem that future increases in population size of the latter may become mediated by availability of the former.

Although evidence suggests that southern right whales breeding off Brazil feed in the region around South Georgia^28^,^30^ historical data indicate that whales can use other foraging grounds in the South Atlantic^53^. Therefore, determining the main feeding ground for the Brazilian population is essential given that the level of climate impacts on the ecosystem may differ among regions. Along Brazilian coast, right whales distributed between Santa Catarina and Bahia states^54^. However, the species was a target for hunting, which occurred illegally until 1973, and the population size declined drastically. Consequently, the distribution was restricted to southern Santa Catarina coast. The population recovery rate for this population is 12% for the period from 1987–2010^55^, and it is expected that the population will probably reoccupy the north of the study area. However, given the influence of climate anomalies on its reproductive success, global changes can be able to jeopardize this recovery and expansion.

Understanding the influence of climate anomalies and variation of food supply on right whale reproductive success may be important for species conservation strategies as they can support decisions about protective measures taken place both in its breeding area off Brazil and in its feeding ground in the Southern Ocean. In addition, krill is an important fishing resource in the south Atlantic sector of the Southern Ocean and, considering the tonnage taken, became the largest fishery in that area^56^. The Commission for the Conservation of Antarctic Marine Living Resources (CCAMLR) is responsible for determining conservation and management actions that take into account the Antarctic marine ecosystem, including those species that depend on krill as a food resource. The overlap between the areas in which krill fisheries operate and feeding areas of krill-dependent species such as southern right whales as well as the influence of climate anomalies on krill availability, underline the importance of CCAMLR including the forecasts of these anomalies in setting long-term objectives for the management of the krill fishery.

The present study suggests that food availability (krill densities) and large-scale climate variables influence the number of calves produced by southern right whales breeding off southern Brazil. This also raises the possibility of using southern right whales in Brazil as a monitoring species to detect changes in krill populations in their foraging areas, a role more typically associated with the land-based predators that occur in the monitored region^57^. We, therefore, highlight the importance of continuing the aerial surveys to estimate the southern right whales number of calves off Brazil. Such data would provide a better understanding of the influence of climate anomalies on southern right whale reproductive success and, consequently, on their recovery in southern Brazil, the second most important breeding ground of the species in the western South Atlantic.

## Additional Information

**How to cite this article**: Seyboth, E. *et al*. Southern Right Whale (*Eubalaena australis*) Reproductive Success is Influenced by Krill (*Euphausia superba*) Density and Climate. *Sci. Rep.* **6**, 28205; doi: 10.1038/srep28205 (2016).

## Figures and Tables

**Table 1:** Aerial survey data on southern right whales on their calving ground along the Brazilian coast obtained from 1997–2013 and data for the variables found as significantly influencing the reproductive success of the species.

**Year**	**Date**	**Number of observers**	**Cow-calf pairs**	**Detrended number of calves**	**Krill (g/m**^**2**^)	**ONI**	**AAO**	**SIAA**
1997	27 Sep	1	7	0.39	31.66	−0.19	−0.20	8.52
1998	23 Sep	2	8	−0.42	38.85	1.69	0.11	8.65
1999	10 Sep	2	8	−2.24	9.69	−1.08	0.84	8.72
2000	8 Oct	2	11	−1.05	2.74	−1.23	0.56	8.77
2001	13 Sep	3	12	−1.86	36.74	−0.58	−0.49	9.03
2002	23 Sep	3	34	18.32	137.03	−0.03	0.24	8.3
2003	12 Sep	3	13	−4.49	84.59	0.76	−0.48	8.84
2004	15 Sep	3	5	−14.30	26.12	0.24	−0.09	8.88
2005	17 Sep	3	18	−3.12	89.42	0.53	0.24	8.8
2006	19 Sep	3	34	11.07	119.11	−0.28	−0.13	8.4
2007	20 Sep	3	29	4.25	61.12	0.36	−0.11	8.71
2008	03 Sep	3	20	−6.56	44.98	−0.97	−0.15	9.29
2009	26 Sep	3	27	−1.37	28.83	−0.38	0.65	9.01
2010	15 Sep	3	25	−5.19	15.05	0.92	−0.30	8.94
2011	10 Sep	3	35	3.00	59	−1.07	0.89	8.86
2012	25 Sep	3	43	9.19	90.11	−0.56	0.04	8.85
2013	13 Sep	3	30	−5.63	61.76	−0.04	0.19	9.26

Krill = krill densities near South Georgia, ONI = Oceanic Niño Index, AAO = Antarctic Oscillation and SIAA = Antarctic sea ice area.

**Table 2:** Cross correlation results for the variables that were indicated as significantly influencing the number of southern right whale calves for the population breeding off southern Brazil.

**Variable**	**Lag (years)**	**R**	**p-value**
Krill	0	0.69	0.002
ONI	6	−0.65	0.03
AAO	7	0.76	0.01
SIAA	0	−0.68	0.002

Krill = krill densities near South Georgia, ONI = Oceanic Niño Index, AAO = Antarctic Oscillation and SIAA = Antarctic sea ice area.

## References

[b1] Doney S. C. . Climate change impacts on marine ecosystems. Ann. Rev. Mar. Sci. 4, 11–37 (2012). 10.1146/annurev-marine-041911-111611 22457967

[b2] Turner J., Bracegirdle T. J., Phillips T., Marshall G. J. & Hosking J. S. An initial assessment of antarctic sea ice extent in the CMIP5 models. J. Climate 26, 1473–1484 (2013).

[b3] Stenseth N. C. . Ecological effects of climate fluctuations. Science 297, 1292–1296 (2002). 12193777 10.1126/science.1071281

[b4] Hodder J. & Graybill M. R. Reproduction and survival of seabirds in Oregon during the 1982–1983 El Niño. The Condor 7, 535–541 (1985).

[b5] Tershy B. R., Breese D. & Alvarez-Borrego S. Increase in cetacean and seabird numbers in Canal de Ballenas during an El Niño-Southern Oscillation event. Mar. Ecol. Prog. Ser. 69, 299–302 (1991).

[b6] Walther G.-R. . Ecological responses to recent climate change. Nature 416, 389–395 (2002). 11919621 10.1038/416389a

[b7] Philander S. G. El Niño, La Niña, and the Southern Oscillation (Academic Press, 1990). 10.1126/science.248.4957.904 17811864

[b8] Trathan P. N. & Murphy E. J. Sea surface temperature anomalies near South Georgia: Relationships with the Pacific El Niño regions. J. Geophys. Res. 108, 1–10 (2003).

[b9] Forcada J., Trathan P. N., Reid K. & Murphy E. J. The effects of global climate in pup production of Antarctic fur seals. Ecology 86, 2408–2417 (2005).

[b10] Forcada J. & Trathan P. N. Penguin responses to climate change in the Southern Ocean. Glob. Change Biol. 15, 1618–1630 (2009).

[b11] Atkinson A., Siegel V., Pakhomov E. A., Jessopp M. J. & Loeb V. A re-appraisal of the total biomass and annual production of Antarctic krill. *Deep-Sea Res*. Pt. I 56, 727–740 (2009).

[b12] Loeb V. . Effects of sea-ice extent and krill or salp dominance on the Antarctic food web. Nature 387, 897–900 (1997).

[b13] Trathan P. N. . In Top predators in marine ecosystems: their role in monitoring and management (eds Boyd I. L., Wanless S. & Camphusen C. J.) Ch 3, 28–45 (Cambridge University Press, 2006).

[b14] Murphy E. J. . Climatically driven fluctuations in Southern Ocean ecosystems. Proc. R. Soc. B 274, 3057–3067 (2007). 10.1098/rspb.2007.1180 PMC2211519 17939986

[b15] Guinet C., Jouventin P. & Georges J.-Y. Long term population changes of fur seals *Arctocephalus gazella* and *Arctocephalus tropicalis* on subantarctic (Crozet) and subtropical (St. Paul and Amsterdam) islands and their possible relationship to El Niño Southern Oscillation. Antarct. Sci. 6, 473–478 (1994).

[b16] Reid K. & Forcada J. Causes of offspring mortality in the Antarctic fur seal, *Arctocephalus gazella*: the interaction of density dependence and ecosystem variability. Can. J. Zool. 83, 604–609 (2005).

[b17] Jenouvrier S. . Effects of climate change on an emperor penguin population: analysis of coupled demographic and climate models. Glob. Change Biol. 18, 2756–2770 (2012). 10.1111/j.1365-2486.2012.02744.x 24501054

[b18] Constable A. J. . Climate change and Southern Ocean ecosystems I: how changes in physical habitats directly affect marine biota. Glob. Change Biol. 20, 3004–3025 (2014). 10.1111/gcb.12623 24802817

[b19] Lockyer C. Body fat condition in northeast Atlantic fin whales, *Balaenoptera physalus*, and its relationship with reproduction and food resource. Can. J. Fish. Aquat. Sci. 43, 142–147 (1986).

[b20] Reeves R. R., Rolland R. & Clapham P. J. (eds) Causes of reproductive failure in North Atlantic right whales: new avenues and research. Report of a workshop held 26–28 April 2000 in Falmouth, Massachusetts. Northeast Fisheries Science Center Reference Document 01–16, 46 p. Available from: National Marine Fisheries Service, 166 Water St., Woods Hole, MA 02543-1026 (2001).

[b21] Greene C. H., Pershing A. J., Kenney R. D. & Jossi J. W. Impact of climate variability on the recovery of endangered North Atlantic right whales. Oceanography 16, 98–103 (2003).

[b22] Ward E. J., Holmes E. E. & Balcomb K. C. Quantifying the effects of prey abundance on killer whale reproduction. Can. J. Fish. Aquat. Sci. 46, 632–640 (2009).

[b23] Williams R. . Evidence of density-dependent changes in body condition and pregnancy rate of North Atlantic fin whales over four decades of varying environmental conditions. ICES J. Mar. Sci., doi: 10.1093/icesjms/fst059 (2013).

[b24] Miller C. A. . Blubber thickness in right whales *Eubalaena glacialis* and *Eubalaena australis* related with reproduction, life history status and prey abundance. Mar. Ecol. Prog. Ser. 438, 267–283 (2011).

[b25] Zieba D. A., Amstalden M. & Williams G. L. Regulatory roles of eptin in reproduction and metabolism. A comparative review. Domest. Anim. Endocrin. 29, 166–185 (2005). 10.1016/j.domaniend.2005.02.019 15927772

[b26] Tormosov D. D. . Soviet catches of southern right whales, *Eubalaena australis,* 1951–1971. Biological data and conservation implications. Biol. Cons. 86, 185–197 (1998).

[b27] Rowntree V. J., Valenzuela L. O., Franco Fragas P. & Seger J. Foraging behavior of southern right whales (*Eubalaena australis*) inferred from variations of carbon stable isotope ratios in their baleen. International Whaling Commission Document SC/60/BRG23 (2008).

[b28] Valenzuela L. O., Sironi M., Rowntree V. J. & Seger J. Isotopic and genetic evidence for culturally inherited site fidelity to feeding grounds in southern right whales (*Eubalaena australis*). Mol. Ecol. 18, 782–791 (2009). 19207250 10.1111/j.1365-294X.2008.04069.x

[b29] Leaper R. . Global climate drives southern right whale (*Eubalaena australis)* population dynamics. Biol. Lett. 2, 289–292 (2006). 17148385 10.1098/rsbl.2005.0431 PMC1618902

[b30] Best P. B., Payne R., Rowntree V., Palazzo J. T. & Both M. C. Long-range movements of South Atlantic right whales *Eubalaena australis*. Mar. Mamm. Sci. 9, 227–234 (1993).

[b31] Seyboth E., Groch K. R., Secchi E. R. & Dalla Rosa L. Habitat use by southern right whales, *Eubalaena australis* (Desmoulins, 1822), in their main northernmost calving area in the western South Atlantic. Mar. Mamm. Sci. 31, 1521–1537 (2015).

[b32] Palazzo J. T. Jr. & Carter L. A. A caça de baleias no Brasil. (AGAPAN, 1983).

[b33] Groch K. R., Palazzo J. T. Jr., Flores P. A. C., Adler F. R. & Fabian M. E. Recent rapid increases in the right whale (*Eubalaena australis*) population off southern Brazil. LAJAM 4, 41–47 (2005).

[b34] Wu Z., Huang N. E., Long S. R. & Peng C. K. On the trend, detrending, and variability of nonlinear and nonstationary time series. Proc. Nat. Acad. Sci. USA 104, 14889–14894 (2007). 17846430 10.1073/pnas.0701020104 PMC1986583

[b35] R Development Core Team. R: A language and environment for statistical computing. Austria: R Foundation for Statistical Computing. Available at: http://www.R-project.org Accessed: June 2015 (2014).

[b36] Fielding S. . Interannual variability in Antarctic krill (*Euphausia superba*) density at South Georgia, Southern Ocean: 1997–2013. ICES J. Mar. Sci., doi: 10.1093/icesjms/fsu104 (2014).

[b37] Loeb V. J., Hofmann E. E., Klinck J. M., Holm-Hansen O. & White W. B. ENSO and variability of the Antarctic pelagic marine ecosystem. Antarct. Sci. 21, 135–148 (2009).

[b38] Liu J., Yuan X., Rind D. & Martinson D. G. Mechanism study of the ENSO and southern high, latitude climate teleconnections. Geophys. Res. Lett. 29, 241–244 (2002).

[b39] Legendre P. & Legendre L. Numerical Ecology. (Elsevier, 1998).

[b40] Braithwaite J. E., Meeuwig J. J., Letessier T. B., Jenner K. C. S. & Brierley A. S. From sea ice to blubber: linking whale condition to krill abundance using historical whaling records. Polar Biol. 38, 1195–1202 (2015).

[b41] George J. C., Druckenmiller M. L., Laidre K. L., Suydam R. & Person B. Bowhead whale body condition and links to summer sea ice and upwelling in the Beaufort Sea. Prog. Oceanogr. 136, 250–262 (2015).

[b42] Berta A., Sumich J. L. & Kovacs K. M. Marine Mammals: Evolutionary Biology (Academic Press, 2005).

[b43] Payne R. Long term behavioral studies of the southern right whale (*Eubalaena australis*). IWC (Special Issue) 10, 161–167 (1986).

[b44] Best P. B., Brandão A. & Butterworth D. S. Demographic parameters of southern right whales off South Africa. J. Cetacean Res. Manag. (Special Issue) 2, 161–169 (2001).

[b45] Cooke J. G., Rowntree V. J. & Payne R. Estimates of demographic parameters for southern right whales (*Eubalaena australis*) observed off Península Valdés, Argentina. J. Cetacean Res. Manag. (Special Issue) 2, 125–132 (2001).

[b46] Cooke J., Rowntree V. & Payne R. Analysis of inter-annual variation in reproductive success of South Atlantic right whales (*Eubalaena australis*) from photo-identifications of calving females observed off Península Valdés, Argentina, during 1971–2000. Paper SC/55/O23 presented at fifty-fifth Annual Meeting of the International Whale Commision, Berlin. Berlin: IWC Scientific Committee (June, 2003).

[b47] Meredith M. P., Murphy E. J., Hawker E. J., King J. C. & Wallace M. I. On the interannual variability of ocean temperatures around South Georgia, Southern Ocean: Forcing by El Niño/Southern Oscillation and the Southern Annular Mode. Deep-Sea Res. Pt. II 55, 2007–2022 (2008).

[b48] Fedulov P. P., Murphy E. & Shulgovsky K. E. Environment-krill relations in the South Georgia marine escosystem. CCAMLR Science 3, 13–30 (1996).

[b49] Murphy E. J. & Reid K. Modelling Southern Ocean krill population dynamics: biological processes generating fluctuations in the South Georgia ecosystem. Mar. Ecol. Prog. Ser. 217, 175–189 (2001).

[b50] Atkinson A., Siegel V., Pakhomov E. & Rothery P. Long-term decline in krill stock and increase in salps within the Southern Ocean. Nature 432, 100–103 (2004). 15525989 10.1038/nature02996

[b51] IPCC. Climate Change 2013: The Physical Science Basis. Contribution of Working Group I to the Fifth Assessment Report of the Intergovernmental Panel on Climate Change (eds Stocker T. F. .) (Cambridge University Press, 2013).

[b52] Cai W. . Increasing frequency of extreme El Niño events due to greenhouse warming. Nature Clim. Change 4, 111–116 (2015).

[b53] IWC (International Whaling Commission). Report of the workshop on the comprehensive assessment of right whales: a worldwide comparison. J. Cetacean Res. Manag. (Special Issue) 2, 1–60 (2001).

[b54] Ellis M. A baleia no Brasil Colonial (Melhoramentos, 1969).

[b55] IWC (International Whaling Commission). Report of the workshop on the assessment of southern right whales, SC/64/Rep5 (2012).

[b56] Nicol S., Foster J. & Kawaguchi S. The fishery for Antarctic krill*–* recent developments. Fish Fish. 13, 30–40 (2012).

[b57] Reid K. The diet of Antarctic fur seals *Arctocephalus gazella* Peters 1875 during winter at South Georgia. Antarct. Sci. 7, 241–249 (1995).

